# Arrhythmogenic Left Ventricular Cardiomyopathy: Genotype-Phenotype Correlations and New Diagnostic Criteria

**DOI:** 10.3390/jcm10102212

**Published:** 2021-05-20

**Authors:** Giulia Mattesi, Alberto Cipriani, Barbara Bauce, Ilaria Rigato, Alessandro Zorzi, Domenico Corrado

**Affiliations:** Department of Cardiac, Thoracic, Vascular Sciences and Public Health, University of Padua, 35128 Padua, Italy; g.mattesi17@gmail.com (G.M.); alberto.cipriani@unipd.it (A.C.); barbara.bauce@unipd.it (B.B.); ilaria.rigato@unipd.it (I.R.); alessandro.zorzi@unipd.it (A.Z.)

**Keywords:** arrhythmogenic cardiomyopathy, left ventricular arrhythmogenic cardiomyopathy, genetics, mutation, differential diagnosis

## Abstract

Arrhythmogenic cardiomyopathy (ACM) is an inherited heart muscle disease characterized by loss of ventricular myocardium and fibrofatty replacement, which predisposes to scar-related ventricular arrhythmias and sudden cardiac death, particularly in the young and athletes. Although in its original description the disease was characterized by an exclusive or at least predominant right ventricle (RV) involvement, it has been demonstrated that the fibrofatty scar can also localize in the left ventricle (LV), with the LV lesion that can equalize or even overcome that of the RV. While the right-dominant form is typically associated with mutations in genes encoding for desmosomal proteins, other (non-desmosomal) mutations have been showed to cause the biventricular and left-dominant variants. This has led to a critical evaluation of the 2010 International Task Force criteria, which exclusively addressed the right phenotypic manifestations of ACM. An International Expert consensus document has been recently developed to provide upgraded criteria (“the Padua Criteria”) for the diagnosis of the whole spectrum of ACM phenotypes, particularly left-dominant forms, highlighting the use of cardiac magnetic resonance. This review aims to offer an overview of the current knowledge on the genetic basis, the phenotypic expressions, and the diagnosis of left-sided variants, both biventricular and left-dominant, of ACM.

## 1. Introduction

Arrhythmogenic cardiomyopathy (ACM) is an inherited heart muscle disease characterized by fibro-fatty replacement of the ventricular myocardium, which predisposes to scar-related ventricular arrhythmias (VAs) and sudden cardiac death (SCD), particularly in the young and athletes [1,2,3]. 

In its original description, the disease was characterized by a predominant involvement of the right ventricle (RV), with left ventricular (LV) lesions occurring later in the history of the disease [4,5]. However, autopsy investigations, studies reporting genotype-phenotype correlations, and the growing use of contrast-enhanced cardiac magnetic resonance (CMR), led to the discovery of biventricular and left-dominant variants, in which the myocardial scar was found earlier and even predominantly in the left ventricle (LV) [6,7,8]. 

The increased awareness that ACM could no longer be considered a disease limited to the RV led to a critical evaluation of the 2010 International Task Force (ITF) criteria, which exclusively addressed the right phenotypic manifestations of ACM [4]. The incomplete understanding of the genetic basis of ACM, the exclusion of tissue characterization findings by contrast-enhanced CMR, and the lack of specific criteria for left-sided ACM were considered the major limitations of the 2010 ITF criteria, that could result in disease underdiagnosis or misdiagnosis [9]. Accordingly, to improve the diagnostic approach to ACM, an International Expert consensus document was recently developed in order to provide upgraded criteria (“the Padua Criteria”) for the diagnosis of the entire spectrum of ACM phenotypes [10]. 

Especially for the left variants, the diagnostic specificity is of outmost importance due to the presence of overlapping phenotypes of other genetic disease, such as cardio-cutaneous syndromes and neuromuscular diseases (NMD), and non-genetic cardiomyopathies, such as dilated cardiomyopathy (DCM), myocarditis, and sarcoidosis [11].

This review aims to offer an overview of the current knowledge on the genetic basis, the phenotypic expressions, and the diagnosis of left-sided variants, both biventricular and left-dominant, of ACM. 

## 2. Left Ventricular Involvement in Arrhythmogenic Cardiomyopathy

In a pathological study of SCD victims, LV involvement was observed to be predominant among those ACM cases diagnosed only at autopsy (87% of the examined hearts; isolated in 17% and with concomitant RV involvement in 70% of cases) [12]. This result underlies the intrinsic limitations of ITF 2010 criteria in diagnosing left ventricular forms of ACM before death and the consequent need of new diagnostic tools and criteria. 

The fibro-fatty scar is the distinctive histological marker of ACM. Both pathological investigations and experimental studies on transgenic animal models demonstrated the genetic basis of the fibro-fatty scar [13,14,15,16,17,18,19,20,21,22,23,24,25,26,27,28,29,30,31]. Numerous chromosomal loci were shown, starting from the first linkage analysis report in 1994 [17,18,19,20,21,22,23,24,25,26,27,28,29,30,31]. Desmosomal alterations reproduced the ACM phenotype in transgenic mice [13,14,15,16]. The distinctive localization in the RV was traditionally labelled as the “triangle of dysplasia”, comprising the RV inflow, apex, and outflow. Transvenous endomyocardial biopsy (EMB) has been comprised of the diagnostic evaluation of ACM since 1994 [32] because it allows an in vivo histologic proof of the pathognomonic disease lesion, i.e., the myocardial necrosis and fibrofatty replacement. Indeed, EMB has been regarded as a major criterion for ACM diagnosis [4,33]. Then, genotype–phenotype studies and the growing use of CMR led to the discovery of biventricular and left-dominant variants. In particular, contrast-enhanced CMR, thanks to its tissue characterization capability resulting from late gadolinium enhancement (LGE) technique, allowed the demonstration of fibro-fatty scars involving the LV [9]. As for the RV, in the LV the fibro-fatty replacement wavefront proceeds from the subepicardial to the subendocardial LV layers, finally resulting in transmural lesion with localized or extensive wall thinning. It has been demonstrated that in left ACM, the scar tissue tends to localize in the inferolateral subepicardial LV wall [6,7,34]. The subendocardium, which predominantly contributes to myocardial thickening, is usually spared [35]. Consequently, differently from the classic right-dominant phenotype, the sensitivity of standard investigations, particularly echocardiography, for the diagnosis of left-dominant forms is low. In particular, the preserved global LV systolic function and the absence of regional wall motion abnormalities explain the normal results of echocardiography. In this scenario, contrast-enhanced CMR represents a fundamental tool for the detection of structural LV involvement. Indeed, while the systolic and regional function can still be unremarkable at CMR cine imaging, contrast-enhanced CMR imaging can show both fat infiltration and presence of fibrosis using T1-weighted sequences and LGE analysis, respectively. The combination of the signal enhancement at both sequences represents the equivalent of the fibro-fatty scar at the pathological study. 

**Figure 1:**
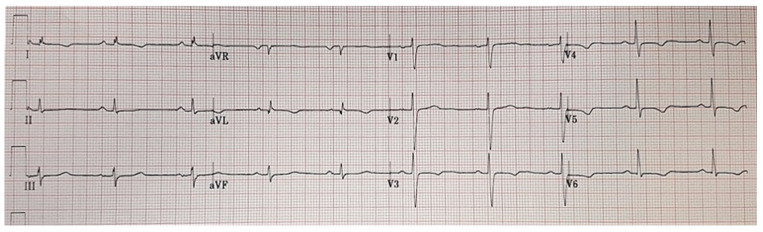
Electrocardiographic features of a patient affected by left-dominant arrhythmogenic cardiomyopathy. Basal electrocardiogram characterized by low QRS voltages in limb leads and negative T-waves in the lateral leads.

**Figure 2:**
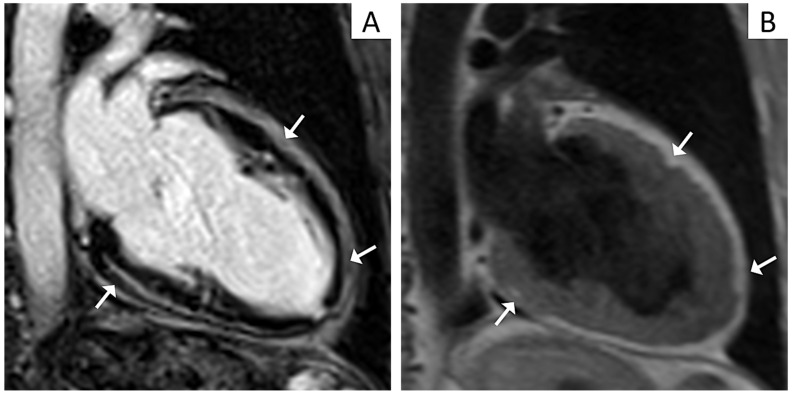
Cardiac magnetic resonance (CMR) imaging features of a patient affected by left-dominant arrhythmogenic cardiomyopathy. Post contrast CMR images in two-chamber view showing extensive late gadolinium enhancement in the form of stria proceeding from the epicardium towards the endocardium in the inferior and anterior walls (Panel **A**, arrows). T1 weighted CMR sequences in two-chamber view evidencing fatty infiltration in the same regions as in Panel **A** (Panel **B**, arrows).

All these features outline an LV phenotype characterized by normal or mildly reduced LV systolic function with no or mild dilatation and extensive non-ischemic LGE predominantly involving the inferolateral segments. In addition to this, other distinctive characteristics are VAs with a right-bundle-branch-block (RBBB) morphology denoting the origin from the LV scar tissue and ECG anomalies such as negative T-waves or flattening in the lateral or inferolateral leads and low QRS voltages (peak-to-peak < 0.5 mV) in the limb leads (Figure 1 and Figure 2) [7,10,36].

## 3. Genetic Basis of Arrhythmogenic Cardiomyopathy

Arrhythmogenic cardiomyopathy generally has an autosomal dominant transmission with incomplete penetrance and variable expression, resulting in heterogeneous clinical manifestations. Less commonly, the disease can manifest with palmoplantar keratoderma and woolly hair and can be transmitted as an autosomal recessive trait (Naxos and Carvajal syndromes). Compound and digenic heterozygosity have been documented with a range of occurrences that varies widely depending on how many genes are sequenced and how missense variants are considered [37,38,39]. Homozygous mutations have also been reported [40]. It is important to note that additional genetic and/or environmental factors may act as modifiers, since the probability of being affected varies between siblings of the same index case. Thus, the role of genetic mutations in ACM pathogenesis should not be simplified as a linear cause–effect relationship in which a certain phenotype corresponds precisely to a particular genetic mutation as well as many other factors, either genetic or non-genetic, should be taken into account. 

The most frequently involved genes in ACM are those encoding for desmosomal proteins, such as plakophilin-2 (*PKP2*), desmoplakin (*DSP*), desmoglein (*DSG2*), desmocollin (*DSC2*), and plakoglobin (*JUP*). Heterozygous mutations determining early protein termination and/or anomalous splicing in *PKP2* are the most common [37]. 

Genes encoding for adherent junctional proteins, such as α-T-catenin (*CTNNA3*) and N-cadherin (*CDH2*), have also arisen as potentially relevant for the pathogenesis of ACM [18,20,22,26,31,41,42,43,44]. 

Desmosomes, adherent junctions, gap junctions, and ion channels make up the area composita, which is found at the intercalated disc level. Because this structure is essential both for cell–cell electromechanical connections and intracellular signaling pathways, mutations of its components not only affect mechanical matching among cardiomyocytes, favoring their separation and consequent cell death and scar formation, but also contribute to the arrhythmogenic pathogenesis of ACM by a purely electrical mechanism [14]. 

Besides the mentioned genes, other non-desmosomal ones have been recognized as pathogenic, such as lamin A/C (*LMNA*), desmin (*DES*), filamin C (*FLNC*), titin (*TTN*), sodium voltage-gated channel alpha subunit 5 (*SCN5A*), phospholamban (*PLN*), transmembrane protein 43 (*TMEM 43*), the cardiac ryanodine receptor-2 (*RYR2*), and transforming grow factor beta-3 (*TGFβ-3*) [24,27,28,29,31,45,46,47,48,49].

Lastly, gene-elusive cases with a typical lower prevalence of positive family history have also been reported [50]. It remains to be established whether they represent genetic variants with low penetrance and high environmental influence, or a primarily monogenic disease. 

Overall, these heterogeneous mutations partially explain the clinical and genetic overlap between ACM and other genetic cardiomyopathies and channelopathies. Indeed, in patients affected by Brugada syndrome, *PKP2* missense mutations have been documented [30].

### 3.1. Genotype-Phenotype Correlations in Left Arrhythmogenic Cardiomyopathy 

#### 3.1.1. Desmosomal Mutations

Desmosomal mutations are more common in subjects who fulfil an ACM diagnosis according to 2010 ITF criteria, supporting that the “classical” right-dominant ACM variant is mainly a disease of the desmosome. However, mutations in these genes have also been associated with “non classical” forms, both biventricular and left-dominant. Advanced stages of right-dominant ACM (often *PKP2* carriers) can show LV involvement at the lateral wall with mildly or moderately reduced LV function [51]. *DSG2* and *DSC* mutations have been related to biventricular variants of ACM [52,53]. *DSP* mutations have been associated to a peculiar phenotype characterized by episodes of acute myocardial injury (chest pain with troponin elevation in the presence of normal coronary arteries, “hot phases”), “burst”-induced LV fibrosis, progressive systolic dysfunction, and high incidence of VAs [54,55]. These forms are often diagnosed as acute myocarditis, thus underlying the poor diagnostic accuracy of classic ACM diagnostic criteria and the need for other diagnostic tools in such cases. Recurrent episodes of acute myocarditis among family members, or a personal history of acute myocarditis combined with a family history of cardiomyopathy or SCD, should raise the suspicion of LV variants of ACM, and tissue characterization and genetic testing should be advised [56,57]. 

#### 3.1.2. Non Desmosomal-Mutations

On the other hand, left-dominant or biventricular forms, severe presentations, as well as atypical findings (e.g., altered conduction, early supraventricular arrhythmias, dystrophinopathy, polytopic VA, or arising from the LV), should suggest non-desmosomal mutations and encourage a broader genetic screening. Among non-desmosomal mutations, the fully penetrant mutation p.S358L in the gene *TMEM43*, endemic to Newfoundland, Canada, was the first to be identified. The p.S358L founder mutation in *TMEM43* has been associated with the most aggressive heterozygous form of ACM, the arrhythmogenic right ventricular cardiomyopathy type V (ARVC-5), characterized by LV involvement and high risk of SCD [58]. Filamin C mutations were originally associated with skeletal myopathy, but isolated cardiac involvement has been documented. These mutations have been associated with LV involvement with systolic dysfunction, marked dilatation and wide fibrosis, and frequent Vas [59]. Furthermore, in *FLNC* as well as *DES* mutations carriers, extensive LV subepicardial circumferential late gadolinium enhancement (LGE)/fibrosis (ring-like pattern) and a higher incidence of SCD has been demonstrated [60,61]. Likewise, in patients with *PLN*-p.Arg14del variants, a distinctive tissue pattern at CMR has been shown with LGE mostly located at the LV postero-lateral wall, subsequent arrhythmias, and LV dysfunction [62]. 

**Figure 3:**
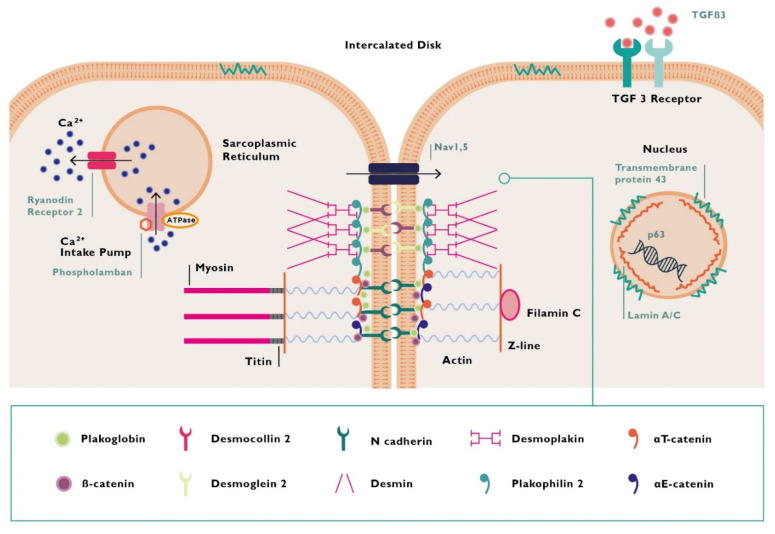
Proteins encoded by mutant genes in arrhythmogenic cardiomyopathy. *TGFB3* = transforming grow factor beta 3.

Figure 3 illustrates encoded proteins associated with ACM. Table 1 summarizes the genetic background of ACM and its correlation with different phenotypes of the disease.

## 4. New Diagnostic Criteria for Arrhythmogenic Cardiomyopathy

These new insights into the heterogeneous genetic mutations and phenotypic manifestations of ACM led to a critical revision of the 2010 ITF criteria, which exclusively targeted RV classical forms and did not include the tissue characterization by contrast enhanced CMR imaging [4,9]. Accordingly, an International Expert consensus document has been recently developed to provide upgraded criteria (“the Padua Criteria”) for the diagnosis of the whole spectrum of ACM phenotypes [10]. The principles behind the Padua Criteria are that:

(1) As for RV forms, the diagnosis of LV variants is multiparametric, and comprises functional and structural ventricular alterations, tissue characterzation results, electrocardiographic (ECG) anomalies, VAs, and familial/genetic factors.
(2) Structural abnormalities can be diagnosed by LGE at contrast enhanced CMR and represents a non-invasive imaging modality for the detection of fibro-fatty scar.
(3) The diagnostic power of phenotypic criteria for left ventricular variants varies in accordance with the disease phenotype, whether biventricular or left-dominant.
(4) When the criteria to diagnose the RV phenotype are met, “phenotypic criteria” such as morpho-functional and structural LV anomalies allows the diagnosis of biventricular variants.
(5) When the RV is not involved, “phenotypic criteria” do not afford sufficient disease-specificity.

In light of this, in patients who meet the traditional 2010 ITF criteria for definite, borderline, or possible ACM, the concomitant presence of morpho-functional and structural LV abnormalities is sufficient to make diagnosis of biventricular forms. Differently, for patients with structural abnormalities (with or without morpho-functional alterations) in whom phenotypic RV alterations cannot be found, the documentation of a positive genotyping is mandatory for making diagnosis of left-dominant variants. Table 2 shows the “Padua criteria” for diagnosis of arrhythmogenic cardiomyopathy [10]. 

Especially for left-dominant variants, the diagnostic specificity is of outmost importance due to the presence of overlapping phenotypes of other genetic and non-genetic disorders that share both genetic and phenotypic features with ACM (i.e., “phenocopies”), such as DCM, myocarditis, sarcoidosis, cardio-cutaneous syndromes, and NMD.

### 4.1. Cardiac Magnetic Resonance Imaging

**Figure 4:**
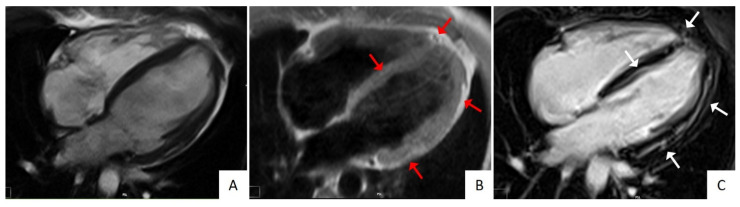
Cardiac magnetic resonance (CMR) imaging features of a patient affected by biventricular arrhythmogenic cardiomyopathy. Cine CMR imaging showing a mildly dilated left ventricle (LV) (Panel **A**) with a slightly reduced systolic function (not showed). T1-weighted CMR images demonstrating fatty infiltration at the right ventricle apex, lateral, and septal LV walls (Panel **B**, red arrows). Post-contrast CMR images showing biventricular LGE in the same locations as in Panel **B** (Panel **C**, white arrows).

In pathological studies, LV involvement was reported in up to 87% of cases [12]. Fibro-fatty changes in ACM affect the LV either diffusely or regionally. Left ventricular structural abnormalities can be visualized as LGE by contrast enhanced CMR, often involving the inferolateral wall and, notably, in the absence of concomitant wall motion abnormalities at CMR cine imaging [6,7,34]. The septum is frequently involved in left-dominant variants, being found in up to 50% of cases, while it is an exceptional finding in right-dominant variants [6]. Fibro-fatty infiltration can be visualized by specific T1-weighted sequences, and results in wall thinning (Figure 4).

While the combination of enhancement at both sequences increases the probability of ACM, the solely LV LGE enhancement is non-specific and present in numerous ischemic or non-ischemic diseases, including myocarditis and sarcoidosis (“phenocopies”). The difficulty in making a differential diagnosis is increased when the also RV is involved. Moreover, sequences for fatty infiltration are not usually included in standard protocols and the fibrofatty scar—the pathognomonic hallmark of the disease—can be missed. Finally, it is important to underline that, even in the presence of the aforementioned CMR alterations, the diagnosis of ACM, whether right, biventricular, or left-dominant, remains multiparametric [63]. Thus, an isolated although indicative CMR finding is not sufficient to make a diagnosis. 

### 4.2. Differential Diagnosis 

Myocarditis can represent a differential diagnostic dilemma with left-sided ACM forms. Subepicardial LV fibrosis can be found in ACM, causing healed myocarditis overdiagnosis. Since it has been reported an higher risk of malignant VAs and SCD in athletes with LV scar [64], this finding should not be considered benign, but it should prompt more in-depth investigations. Differential diagnosis between left ACM and myocarditis may be difficult because myocarditis can be a clinical presentation of some patients with ACM (“hot phases”) [65]. On the other hand, myocarditis can show VAs with a RBBB morphology, denoting the origin from the LV, and ECG anomalies such as negative T-waves and low QRS voltages [66]. In this context, molecular genetic testing with demonstration of AC-gene mutation—typically DSP mutations—is of outmost importance to exclude left-sided forms of ACM [67]. 

**Figure 5:**
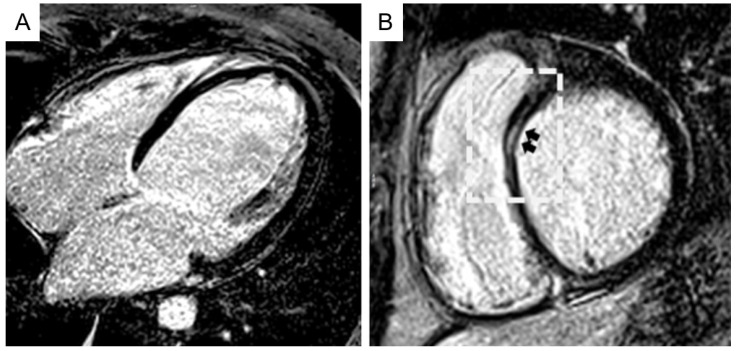
Cardiac magnetic resonance (CMR) imaging features of a patient affected by dilated cardiomyopathy. Post-contrast long-axis (**A**) and short-axis (**B**) CMR views evidencing a severely dilated left ventricle cavity and myocardial late gadolinium enhancement (black arrows), limited to the anteroseptal region (boxed area). Modified from ref [70].

Dilated cardiomyopathy is another tricky mime of left ACM. The resemblance between the two cardiomyopathies encompasses etiological, clinical, and imaging aspects. Desmosomal gene mutations have been documented also in DCM patients [68]. Because DCM can express in milder forms, especially in relatives, the spectrum of LV involvement in DCM ranges from limited scars as assessed by CMR imaging to severely dilated and impaired LV [69]. In this regard, the “hypokinetic non-dilated cardiomyopathy”, which has been considered as a less expressed phenotype of DCM, characterized by an extensive scarring of the LV scarcely affecting the LV systolic function, seems to belong more to the ACM left variants rather than to the DCM spectrum. Indeed, at variance with ACM, in DCM the degree of LV dysfunction does not correlate with the amount of LGE (Figure 5) [70]. Finally, one-third of DCM cardiomyopathies display an arrhythmogenic propensity with a poorer prognosis and an increased SCD risk in analogy with ACM [71]. With the knowledge of these similarities, genetic testing is often needed to reach the correct diagnosis between the two entities.

Cardiac sarcoidosis is associated with life-threatening arrhythmias and heart failure due to the presence of infiltrative granulomas and fibrosis [72]. The RV free wall is interested in up to 40% of cases, while at the LV level, the septum and the free wall are the most common locations [73]. These features lead to difficulties in the differential diagnosis with biventricular and left-dominant ACM. However, some details can help the diagnostic assessment. First, in sarcoidosis cardiac disease is usually observed in the context of a multiorgan disorder interesting the lungs, skin, liver, and eyes, while isolated forms are rarer. Second, when the granulomas infiltrate the basal interventricular septum, bundle branch block and atrioventricular block can appear consequently, whereas they are usually absent in ACM. Finally, CMR imaging can offer some clues useful for distinguishing sarcoidosis from ACM, as described below. In sarcoidosis, myocardial granulomas can be evidenced at post-contrast images as intramural, patchy LGE, mostly located in the basal lateral wall, not related to a coronary distribution territory and responsive to immunosuppressive therapy. An intensive signal at RV insertion points extending into the septum and the RV (“the hook sign”) is also associated with a high probability of sarcoidosis. Moreover, extracardiac findings can be documented. The combination with positron emission tomography can finally highlight fluorodeoxyglucose uptake, which is indicative of active inflammatory lesions [74]. 

Cardiomyopathies associated with neuromuscular disorders (i.e., Duchenne and Becker distrophinopathies) can be undistinguishable from left-dominant ACM, especially when the cardiac disorder occurs in isolation. As it is observed in ACM, the myocardial scar as evidenced by LGE is typically located at the lateral wall with a subepicardial distribution, and acts as a substrate for ventricular tachyarrhythmias, which carry a risk of SCD [75,76]. There is also an overlap in the genetic background, since LMNA and FLNC gene mutations can occur not only in left ACM but also in muscular dystrophies, and the “hypokinetic, non-dilated cardiomyopathy” is the phenotypic appearance of the disease determined by these mutations [46,77]. 

## 5. Prognostic Role of LV Involvement and Genetic Mutations 

Left ventricular involvement, both isolated and combined with a RV disease, demonstrated by clinical, echocardiographic, and invasive exploration, proved to be a predictor of adverse prognosis [78,79,80,81]. However, it has been demonstrated that if the aim is to detect the LV involvement in ACM, the sole estimation of the LV function is not enough. Conversely, contrast-enhanced CMR raises the diagnostic sensitivity allowing the documentation of LV fibrosis, which spare the subendocardial layers and, consequently, do not impair the regional or global LV function [35]. The identification of the LV involvement in the form of LGE has significant implications in terms of prognosis and thus of prophylactic treatment [82]. Indeed, the indication for implantable cardiac defibrillator (ICD) implantation may be considered even in the absence of a severely impaired LV function [78]. In particular, risk stratification in ACM distinguishes three risk categories (“high”, “moderate”, and “low”) with different levels of recommendation to ICD implantation [78]. “High-risk” subjects (class I recommendation for ICD implantation) are those who have a history of aborted SCD or hemodynamically unstable VT or who have severely depressed LV function, either right or left or of both ventricles [78]. Patients with an “intermediate risk” (class IIa recommendation for ICD implantation) are those with major risk factors, like syncope, non-sustained ventricular tachycardias, or moderate disfunction of the right or left or of both ventricles [78]. The inclusion of moderate levels of dysfunction into this category reflects the awareness that the risk of VAs and SCD cannot exclusively be estimated by the LV function since the presence of scars in ACM may not affect the LV performance, but can still trigger adverse arrhythmic events. 

Genotyping is not only useful for the diagnostic workup, but it can also allow prognostic stratification of ACM patients, particularly in terms of the risk prediction of SCD or heart failure. As mentioned above, some genetic mutations have been associated with phenotypes with a higher risk of VAs and SCD. Mutations of FLNC, DSP, and PLN genes predispose to LV lesions and heart failure [6,37,46]. Male carriers of the TMEM43 p.S358L founder mutation show a higher disease penetrance and arrhythmic risk [23].

## 6. Conclusions

Left ventricular arrhythmogenic cardiomyopathy is an emerging entity that has probably been underestimated in the past. Clinical, electrocardiographic, and arrhythmic features should raise the diagnostic suspicion regardless normal echocardiography, and prompt more in-depth investigations. Cardiac magnetic resonance improves the diagnostic sensitivity because it allows the detection of subepicardial/mid-mural LV scars. Myocardial scar is a non-specific finding, and accurate medical history, genetic testing, and clinical family screening can highlight the genetic basis of the disease. The LV involvement as evidenced by cardiac magnetic resonance and/or genetic testing is valued not only for diagnostic purposes but also because it can stratify the arrhythmic risk of ACM patients. The National ARVC Data Registry and Bio Bank, (https://clinicaltrials.gov/ct2/show/record/NCT01804699, accessed on 13 April 2021) will provide data on phenotype–genotype correlations of heterogeneous genetic backgrounds.

## Figures and Tables

**Table 1:** Summary table for the genetic background of arrhythmogenic cardiomyopathy and its correlation with different phenotypes of the disease.

Gene	Encoded Protein	Chromosomal Locus	Mode of Transmission	Reference	Predominately Affected Ventricle	Notes
**Desmosomal**						
***JUP***	Junction plakoglobin	17q21.2	AD/AR also reported	McKoy et al. [18]	RV, biventricular	AR form: Cardiocutaneous syndrome (Naxos)
***DSP***	Desmoplakin	6p24.3	AD/AR also reported	Norgett et al. [19]	LV, biventricular	AR form: Cardiocutaneous syndrome (Carvajal)
***PKP2***	Plakophillin-2	12p11.21	AD/AR also reported	Gerull et al. [20]	RV, biventricular	classic RV phenotype
***DSG2***	Desmoglein-2	18q12.1	AD/AR also reported	Awad et al. [21]	classic RV phenotype, biventricular	frequent LV involvement
***DSC2***	Desmocollin-2	18q12.1	AD/AR also reported	Syrris et al. [22]	RV, biventricular	AR Cardiocutaneous form
**Non desmosomal**						
***TMEM43***	Transmembrane protein 43	3p25.1	AD	Merner et al. [23]	RV, biventricular	Newfoundland founder variant, SCD
***LMNA***	Lamin A/C	1q22	AD	Quarta et al. [24]	LV, biventricular	Overlapping syndrome (DCM, Lipodystrophies, Myopathies)
***DES***	Desmin	2q35	AD	Hedberg et al. [25]	LV, biventricular	Overlapping syndrome (DCM and HCM, early conduction disturbances)
***CTNNA3***	Alpha T-catenin	10q21.3	AD	Van Hengel et al. [26]	RV, biventricular	Low penetrance
***PLN***	Phospholamban	6q22.31	AD	Van der Zwaag et al. [27]	LV, biventricular	Founder mutation in Netherlands. High SCD risk.
***TGFB3***	Transforming grow factor beta 3	14q24.3	AD	Beffagna et al. [28]	RV	
***TTN***	Titin	2q31.2	AD	Taylor et al. [29]	RV, LV, biventricular	Overlapping syndrome (early conduction disturbances, AF, DCM)
***SCN5A***	Sodium voltage-gated channel alpha subunit 5		AD	Cerrone et al. [30]	LV, biventricular	Overlap syndrome (BrS, LQTS Type 3, AF)
***CDH2***	Cadherin C		AD	Mayosi et al. [31]	RV, biventricular	

AD = autosomal dominant; AF = atrial fibrillation; AR = autosomal recessive; BrS = Brugada syndrome; DCM = dilated cardiomyopathy; HCM = hypertrophic cardiomyopathy; LQTS = long QT Syndrome; LV = left ventricle; RV = right ventricle; SCD = sudden cardiac death.

**Table 2:** “Padua criteria” for diagnosis of arrhythmogenic cardiomyopathy.

Category	Right Ventricle	Left Ventricle
I. *Morpho-functional ventricular abnormalities*	By echocardiography, CMR or angiography: *Major* Regional RV akinesia, dyskinesia, or bulging, plus, one of the following: - global RV dilatation (increase of RV EDV according to the imaging test specific nomograms) - global RV systolic dysfunction (reduction of RV EF according to the imaging test specific nomograms) *Minor* Regional RV akinesia, dyskinesia, or aneurysm of RV free wall	By echocardiography, CMR or angiography: *Minor* Global LV systolic dysfunction (depression of LV EF or reduction of echocardiographic global longitudinal strain), with or without LV dilatation (increase of LV EDV according to the imaging test specific nomograms for age, sex, and BSA) *Minor* Regional LV hypokinesia or akinesia of LV free wall, septum, or both
II. *Structural myocardial abnormalities*	By CE-CMR: *Major* Transmural LGE (stria pattern) of ≥1 RV region(s) (inlet, outlet, and apex in 2 orthogonal views) By EMB (limited indications): *Major* Fibrous replacement of the myocardium in ≥1 sample, with or without fatty tissue	By CE-CMR: *Major* LV LGE (stria pattern) of ≥1 Bull’s Eye segment(s) (in 2 orthogonal views) of the free wall (subepicardial or midmyocardial), septum, or both (excluding septal junctional LGE
III. *Repolarization abnormalities*	*Major* Inverted T waves in right precordial leads (V1, V2, and V3) or beyond in individuals with complete pubertal development (in the absence of complete RBBB) *Minor* Inverted T waves in leads V1 and V2 in individuals with completed pubertal development (in the absence of complete RBBB) Inverted T waves in V1, V2, V3 and V4 in individuals with completed pubertal development in the presence of complete RBBB.	*Minor* Inverted T waves in left precordial leads (V 4–V 6) (in the absence of complete LBBB
IV. *Depolarization abnormalities*	*Minor* Epsilon wave (reproducible low amplitude signals between end of QRS complex to onset of the T wave) in the right precordial leads (V1 to V3) Terminal activation duration of QRS ≥ 55 ms measured from the nadir of the S wave to the end of the QRS, including R’, in V1, V2, or V3 (in the absence of complete RBBB)	*Minor* Low QRS voltages (<0.5 mV peak to peak) in limb leads (in the absence of obesity, emphysema, or pericardial effusion)
V. *Ventricular arrhythmias*	*Major* Frequent ventricular extrasystoles (>500 per 24 h), non-sustained or sustained ventricular tachycardia of LBBB morphology *Minor* Frequent ventricular extrasystoles (>500 per 24 h), non-sustained or sustained ventricular tachycardia of LBBB morphology with inferior axis (“RVOT pattern”)	*Minor* Frequent ventricular extrasystoles (>500 per 24 h), non-sustained or sustained ventricular tachycardia with a RBBB morphology (excluding the “fascicular pattern”)
VI. *Family history/genetics*	*Major* ACM confirmed in a first-degree relative who meets diagnostic criteria ACM confirmed pathologically at autopsy or surgery in a first degree relative Identification of a pathogenic or likely pathogenetic ACM mutation in the patient under evaluation *Minor* History of ACM in a first-degree relative in whom it is not possible or practical to determine whether the family member meets diagnostic criteria Premature sudden death (<35 years of age) due to suspected ACM in a first-degree relative ACM confirmed pathologically or by diagnostic criteria in a second-degree relative	

ACM = arrhythmogenic cardiomyopathy; BSA = body surface area; EDV = end diastolic volume; EF = ejection fraction; ITF = International Task Force; LBBB = left bundle-branch block; LGE = late gadolinium enhancement; LV = left ventricle; RBBB = right bundle-branch block; RV = right ventricle; RVOT = right ventricular outflow tract. From Corrado et al. [10].

## Data Availability

Data available in a publicly accessible repository.
